# Lung lesion score system in cattle: proposal for contagious bovine pleuropneumonia

**DOI:** 10.1007/s11250-017-1409-2

**Published:** 2017-09-27

**Authors:** Andrea Di Provvido, Giovanni Di Teodoro, Geoffrey Muuka, Giuseppe Marruchella, Massimo Scacchia

**Affiliations:** 1 Istituto Zooprofilattico Sperimentale dell’Abruzzo e del Molise, OIE Reference Laboratory for Contagious Bovine Pleuropneumonia, Campo Boario, 64100 Teramo, Italy; 2 Faculty of Veterinary Medicine, University of Teramo, Loc. Piano d’Accio, 64100 Teramo, Italy; 3 Ministry of Fisheries and Livestock, Veterinary Department, Central Veterinary Research Institute, Lusaka, Zambia

**Keywords:** Cattle, Contagious bovine pleuropneumonia, Pathology, Lung lesion scoring systems

## Abstract

Contagious bovine pleuropneumonia (CBPP) is a severe infectious disease caused by *Mycoplasma mycoides* subsp. *mycoides*. The peculiar pathological features of CBPP make desirable the assessment of ad hoc score methods to grade the disease in the affected animals. Thus, the present work aims to assess a new lung score system for CBPP. Our results indicate that the present score system strongly correlates with that previously published by Turner and could be effectively used in CBPP-affected animals.

## Introduction

Respiratory diseases commonly occur in all animal species and notably impact upon the profitability of farms, both directly (mortality, poor daily weight gain) and indirectly (costs for antimicrobial drugs, vaccines, veterinary cares, etc.). The etiology of respiratory diseases is usually considered multifactorial and involves a number of risk factors, which can be grouped and summarized as follows:

- management—overcrowding, commingling with animals from multiple sources, environmental conditions such as temperature, humidity, dust levels;
- individual factors—age, breed, immune status, concurrent diseases;
- pathogens—viruses, bacteria, parasites (Taylor et al. 2010).

Considering that the correct diagnosis is crucial for the efficient treatment and/or prevention of respiratory diseases. Pathological findings observed at necropsy are extremely useful to make a rapid, presumptive diagnosis, as well as to drive further laboratory investigations. In addition, postmortem inspections allow “scoring” of lung lesions, such assessment being essential in order to evaluate the efficacy of the therapeutic and/or preventive measures undertaken.

The assessment of reliable, standardized lung score systems, which can easily be carried out even under field conditions (e.g., at the slaughterhouse), has been required for a long time to assess the health status of animal populations. A number of methods have been proposed also for bovine respiratory diseases, including contagious bovine pleuropneumonia (CBPP; Table 1).

**Table 1:** Score systems currently available to assess lung lesions in cattle

Authors	Goal	Scoring system
Thomson et al. (1975).	To investigate risk factors for pneumonic pasteurellosis	Visual inspection of the lungs. Score 0–5 for each lung lobe. Maximum value = 35
Jericho and Langford (1982); Groom et al. (1988).	To evaluate the efficacy of aerosol vaccination with *Pasteurella haemolytica*	Visual inspection of the lungs. The score was expressed as the estimated percentage of pneumonic tissue
Thomas et al. (1989).	To study the pathogenesis of bovine pneumonic pasteurellosis	Visual inspection of the lungs. The score was expressed as the percentage of affected dorsal lung surface
Hubschle et al. (2003).	To evaluate a vaccine for CBPP	Presence/absence of CBPP-induced lung lesions
Hanzlicek et al. (2010).	To study the clinic-pathological kinetic of pneumonia caused by *Mannheimia haemolytica*	Palpation, visual inspection, and measurement of lesion cubic area. The score was calculated as the pneumonic percentage of each lobe multiplied for a lobe-specific corrective factor
Leruste et al. (2012).	To evaluate the relationship between clinical signs and lung lesions at slaughter	Visual inspection of the lungs. Score 0–3 for the entire lung parenchyma
White et al. (2012); Amrine et al. (2013).	To study the relationship among clinical signs, behavioral changes, and lung lesions in *Mycoplasma bovis*-infected calves	The score was calculated by multiplying the percentage of consolidation in each lobe by the proportion of the total lung that each lobe represented
Turner (1961).	To evaluate the efficacy of vaccination against CBPP	Two points for acute or necrotic lesions; one point for healing process. Such scores were multiplied by a severity factor (1 to 3) according to the size of the lesions. Possible scores = 0, 1, 2, 4. and 6

*CBPP* contagious bovine pleuropneumonia

**Fig. 1:**
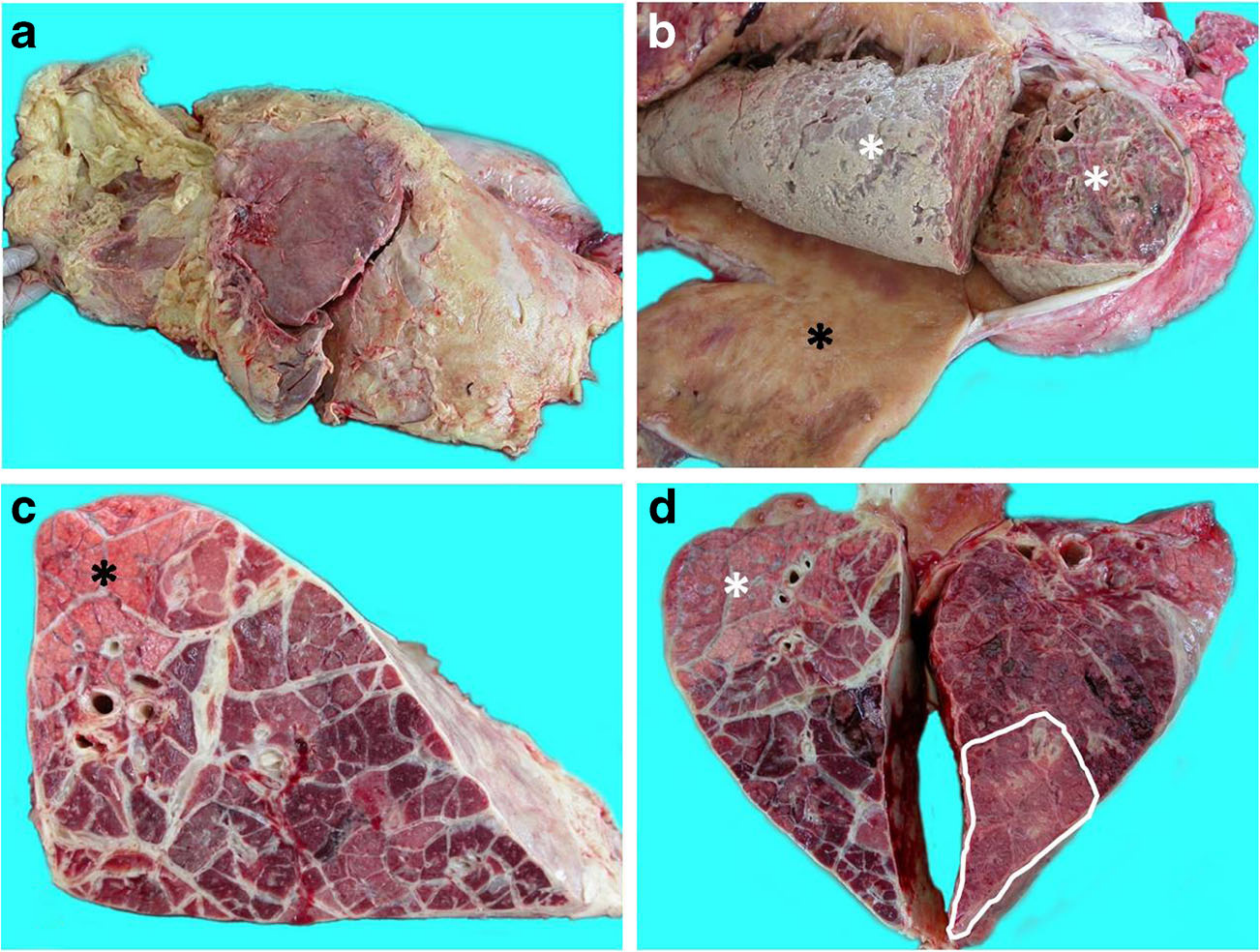
Main pathological findings in CBPP-affected cattle. **a** The entire left lung is covered by a thick layer of fibrin (fibrinous pleurisy). **b** Enormous sequestrum consisting of necrotic parenchyma (white asterisk) surrounded by a thick fibrotic capsule (black asterisk). **c** On cut section, a small portion of the healthy lung is seen (black asterisk), while the remaining surface shows red to gray hepatization of lobules and thickening of interlobular septa (so-called marbling appearance of the lung). **d** In this case, a large area of necrosis is seen (bordered by a white line). Healthy parenchyma (white asterisk) and hepatization of lobules are also observed

CBPP is a severe infectious disease caused by *Mycoplasma mycoides* subsp. *mycoides* (*Mmm*), which is still widespread and of major economic significance in sub-Saharan Africa (Anonymous 2014; Thiaucourt et al. 2004). CBPP-induced lesions vary during the course of the disease, as shown in Fig. 1. The peculiar pathological features of CBPP make the assessment of ad hoc lung lesion score systems desirable. In this respect, Turner (1961) developed a specific method to score CBPP lesions (Table 2). Although widely used, we consider that Turner’s scoring system shows some “weak points” (e.g., it does not consider pleurisy), which would be hopefully overcome.

On the basis of the above considerations, the main goals of the present paper are to assess a new lung lesion score system for CBPP and to compare it with Turner’s method.

## Materials and methods

A total of 98 “zebuine” cattle, experimentally infected with *Mmm* by intubation (*n* = 56) or by contact (*n* = 42), have been included in the present study. Experimental investigations were carried out in Namibia (2004) and Zambia (2016), according to the animal testing regulations applied therein.

**Fig. 2:**
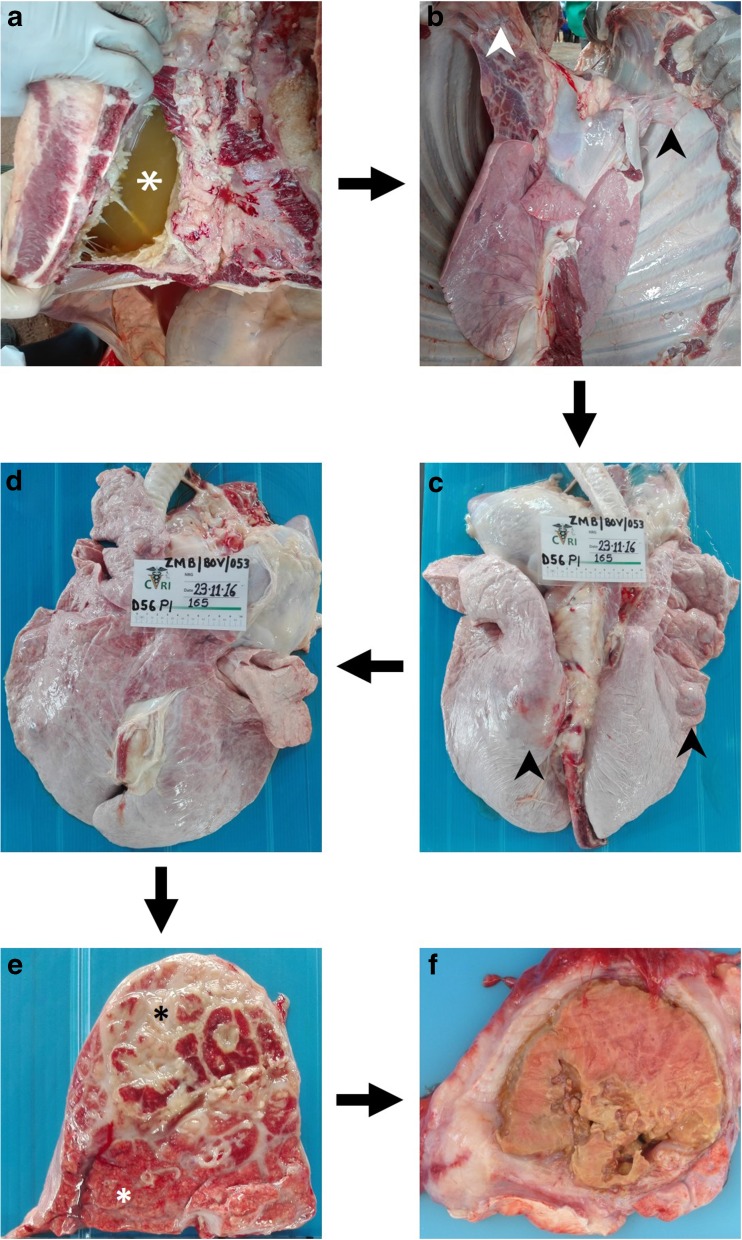
Postmortem inspection of the thoracic cavity and lungs. **a** The thoracic cavity is evaluated for the presence of pleural effusion. In this picture, many liters of yellowish, turbid, fibrinous exudate are seen (asterisk). **b** After the complete opening of the chest, the thoracic cavity is inspected to appreciate the presence, if any, of pleural adhesion (black and white head arrows) and to carefully remove both lungs. **c** The dorsal surface of both lungs is visually inspected to observe the presence, the extent, and the main features of pleurisy. Then, both lungs are entirely and carefully palpated to notice possible *foci* of consolidation. In this picture, large lesions modifying the profile of the lungs (head arrows) can be easily detected. **d** Likewise, the ventral surface of both lungs is subsequently inspected. **e** Each lesion is assessed on cut section to evaluate its features and extent. In this picture, about 50% of the section is affected by fibrosis (black asterisk). At the bottom, “healthy”, pinky lobules are seen (white asterisk). **f** A large sequestrum, surrounded by a thick capsule and affecting the entire cut section, is observed

Cattle were culled and subjected to in-depth postmortem inspection. The lungs were first observed in situ, after opening the thoracic cavity, then removed and carefully inspected physically by skilled veterinarians (Fig. 2).

**Fig. 3:**
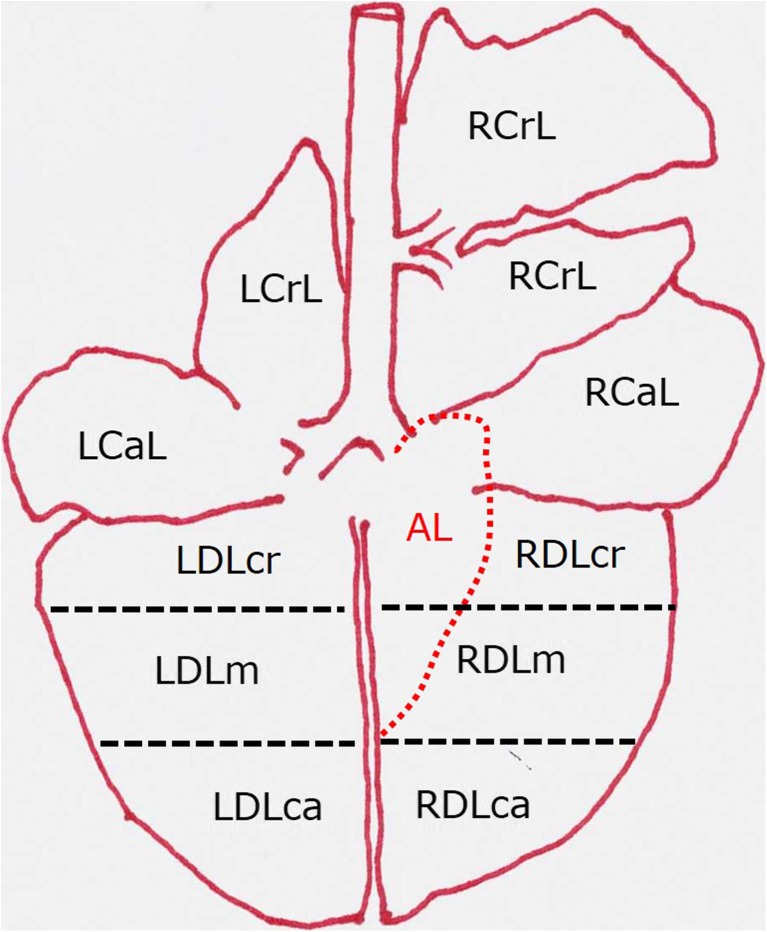
Subdivision of the bovine lungs in areas—graphical representation. RCrL right cranial lobe, RCaL right cardiac lobe, RDL right diaphragmatic lobe, AL accessory lobe, lying on the ventral surface of the right lung and herein bordered by a red dashed line, LCrL left cranial lobe, LCaL left cardiac lobe, LDL left diaphragmatic lobe. Each diaphragmatic lobe is further subdivided into three areas (cranial, medial, and caudal), herein bordered by black dashed lines

As shown in Fig. 3, the lungs were subdivided into 11 “areas”, which were similar in volume. At first, the lungs were visually inspected to notice the presence, if any, and the extent of acute (fibrinous) and/or chronic (adhesive) pleurisy. The presence of pleurisy scored 1 point *per* each affected area; as a result, the pleurisy score ranged between 0 and 11.

Then, the lungs were evaluated by palpation, and all *foci* of consolidation were assessed on cut section; to that aim, the entire thickness of the lung was cut perpendicularly to its main axis. Each lesion was evaluated at its largest point and photographed. Pathological features (red and gray hepatization, necrosis, sequestra, fibrosis) were recorded in each area and scored as follows:

- 0 points = absence;
- 1 point = < 25% of the cutting section;
- 2 points = 25–50% of the cutting section;
- 3 points = 50–75% of the cutting section;
- 4 points = > 75% of the cutting section.

Small-size sequestra (“microsequestra”, < 1.5 cm in diameter) did not fit in the above categories and were scored on the basis of their number:

- 1 to 5 microsequestra = 1 point;
- 6 to 10 microsequestra = 2 points;
- > 11 microsequestra = 3 points.

**Fig. 4:**
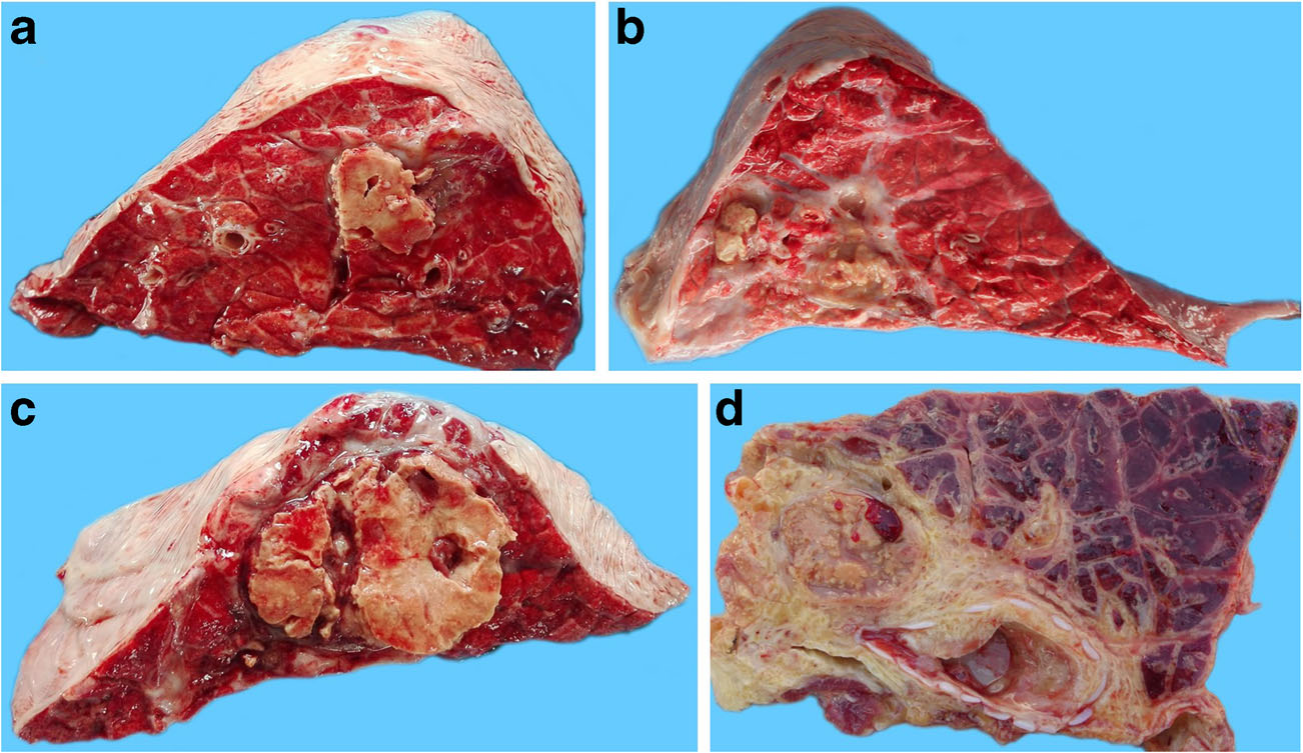
Explanatory examples of CBPP scores following the system proposed herein. **a** Score 1 = a small sequestrum, about 2 cm in diameter, cover less than 25% of the entire cut section. **b** Score 2 = two sequestra are seen, along with the fibrosis of interlobular septa. Diseased parenchyma amounts to about 35–40% of the cut section. **c** Score 3 = a large sequestrum occupies about two thirds of the cut section. **d** Score 4 = about 75% of the entire cut section is affected by red hepatization (3 points). A sequestrum is also detected, covering about the 25% of the entire surface (1 point)

Using this method, the maximum possible score amounted to 55 (explanatory examples are provided in Fig. 4). All recorded data were reported in a file format of Microsoft Excel, thus being available for statistical processing.

In addition, the lungs under study were scored according to Turner (1961), as summarized in Table 2; Turner’s scores were also reported in a file format of Microsoft Excel.

**Table 2:** CBPP score system proposed by Turner (1961)

Pathological features		Multiplying severity factor (size of lesion)		
		1–5 cm	6–20 cm	> 20 cm
Acute-to-necrotic lesions	2 points	×1	×2	×3
*Total score =*		*2*	*4*	*6*
Fibrosis, resolving lesions	1 point	×1	×2	×3
*Total score =*		*1*	*2*	*3*

As shown in the Table, this system allots 1 point if only fibrotic/resolving lesions are observed, while 2 points are assigned to acute-to-necrotic lesions. Such scores are multiplied for a severity factor, which ranges between 1 and 3 and depends on the size of lesion. Therefore, the maximum possible score is 6 points

Finally, the correlation (*Pearson*’s correlation coefficient) between the two lung score systems was calculated by means of the Analysis Toolpak add-in in Microsoft Excel.

## Results

The pathological assessment of each animal was rapid. The scores resulting from both methods are reported in Table 3. The features and the severity of CBPP lesions were highly variable. Pleurisy was observed in 81 cattle, it was acute (fibrinous pleurisy, *n* = 17) or chronic (pleural adhesion, *n* = 64), most commonly affected the diaphragmatic lobes and overlaid pneumonic *foci*. Acute/subacute lung lesions (red and gray hepatization, respectively) were detected in 15 animals, while areas of pulmonary necrosis were seen in 28 cattle. In chronic cases, fibrosis (*n* = 17), sequestra (*n* = 42), and microsequestra (*n* = 21) were detected.

**Table 3:** Comparison of data obtained by the two different lung score systems

ID	Turner	New	ID	Turner	New	ID	Turner	New	ID	Turner	New
1	2	2	26	4	5	51	4	7	76	3	2
2	2	1	27	2	2	52	4	5	77	0	1
3	4	4	28	4	6	53	1	3	78	1	4
4	0	4	29	2	2	54	6	12	79	6	7
5	4	8	30	2	3	55	6	10	80	6	16
6	6	10	31	0	3	56	6	30	81	6	8
7	0	1	32	2	3	57	6	21	82	6	11
8	1	8	33	4	6	58	0	2	83	6	6
9	4	5	34	2	4	59	2	2	84	6	26
10	4	2	35	2	5	60	6	15	85	6	25
11	4	6	36	4	5	61	6	10	86	6	16
12	0	2	37	2	1	62	6	20	87	4	9
13	2	5	38	2	2	63	6	25	88	6	20
14	2	1	39	4	6	64	2	7	89	6	30
15	4	3	40	2	1	65	4	5	90	6	30
16	2	2	41	4	6	66	2	4	91	6	41
17	2	1	42	2	3	67	2	5	92	6	30
18	2	1	43	0	2	68	6	55	93	6	29
19	0	2	44	4	6	69	4	4	94	6	31
20	2	2	45	2	3	70	6	25	95	6	25
21	6	15	46	4	5	71	6	26	96	6	15
22	0	1	47	4	4	72	4	7	97	6	25
23	2	6	48	2	4	73	6	25	98	6	30
24	4	13	49	2	3	74	6	30			
25	0	1	50	4	3	75	6	55			

*ID* identification number of each animal included in the present study, *Turner* CBPP lung score system proposed by Turner (1961), *New* new proposal for CBPP lung score system

As a consequence, scores also largely varied, ranging between 0 and 6 and 1–55 (Turner’s and “new CBPP score system”, respectively). The two scoring systems appeared positively and strongly correlated, the *Pearson*’s coefficient being 0.71.

## Discussion

Postmortem assessment of lesions provides valuable information to manage the health status in livestock. Up to date, that proposed by Turner is the only suitably developed method to score CBPP lesions. Although useful and easy to perform under field conditions, this method shows some limitations, as it does not cater to all categories of CBPP lesions.

First of all, the Turner scoring system does not consider pleurisy which, in our experience, could be the only relevant lesion at the time of postmortem inspection, mostly in vaccinated and/or experimentally infected and/or naturally recovering cattle. Moreover, the Turner system only partially distinguishes CBPP lesions on the basis of their features (i.e., the stage of development) and size; for example, an animal with a large sequestrum (> 20 cm in diameter) gets the maximum score (6 points), in the same way as one with a diffuse, bilateral, acute-to-subacute pleuropneumonia. Scoring lung lesions according to their size (expressed in cm) could also raise some concerns, considering that cattle size can vary widely among breeds.

Aiming to “mitigate” such limitations, we designed the present lung lesion score system, which summarizes those previously published by other authors and is herein considered useful to quantify CBPP lesions. As an example, our system partly overlaps the “consolidation lung lesion score”, which also splits the lungs in “areas” and is currently recommended by the European Pharmacopeia for porcine pleuropneumonia (Sibila et al. 2014).

Although apparently complicated, the present method requires a few minutes to assess individual animals; at the same time, collected data provide in-depth information about the stage (acute-to-chronic) and the severity of the disease. In addition, disaggregated data—recorded in a file format of Microsoft Excel—could allow for a number of additional considerations, useful to evaluate the effect of therapeutic/preventive measures.

The natural challenge in cattle by the in-contact method is the only way to control the potency of a vaccine against CBPP (Anonymous 2014). Therefore, the assessment of well-detailed lung score systems could fill such gaps. In this respect, further investigations are needed to relate CBPP scores—obtained with this and/or other methods—with clinical, productive, and diagnostic parameters.

Experience has shown that taking pictures (detailed and of good quality) from all investigated cattle proved to be quite time-consuming, challenging, not easily standardized, and often unfeasible (Sibila et al. 2014). Nevertheless, it is worth planning such an activity, which allows a more precise, as well as retrospective evaluation of each CBPP-affected animal. In addition, these pictures could be available for “digital scoring” of lesions.

In conclusion, the present scoring method strongly correlates with that proposed by Turner (1961), which was the only CBPP-specific lung score system available, repeatedly used in experimental investigations to assess the efficacy of vaccines anti-CBPP. We consider that our lung score system adequately details the pathological findings observed in CBPP-affected cattle and could be effectively used, mostly in experimental studies, to evaluate the efficacy of preventive or therapeutic measures for CBPP.
